# Myiasis During Adventure Sports Race

**DOI:** 10.3201/eid1001.020825

**Published:** 2004-01

**Authors:** Mikko Seppänen, Anni Virolainen-Julkunen, Iiro Kakko, Pekka Vilkamaa, Seppo Meri

**Affiliations:** * Helsinki University Central Hospital, Helsinki, Finland; † University of Helsinki, Helsinki, Finland; ‡ Sports Institute of Eerikkilä, Tammela, Finland; § Finnish Museum of Natural History, Helsinki, Finland

**Keywords:** Screwworm infection, myiasis, sports, travel, leisure activities, wounds and injuries

## Abstract

Travelers who have visited tropical areas may exhibit aggressive forms of obligatory myiases, in which the larvae (maggots) invasively feed on living tissue. The risk of a traveler’s acquiring a screwworm infestation has been considered negligible, but with the increasing popularity of adventure sports and wildlife travel, this risk may need to be reassessed.

## Case Report

In November 2001, a 41-year-old Finnish man, who was participating in an international adventure sports race in Para (a jungle area in Brazil), tripped at night over a loose rock while he was riding a bicycle. He received a bruised, lacerated wound on his left dorsal antebrachium, which he quickly wiped with paper. The remaining gravel, dirt, and three unidentified winged insects were removed, and the wound was thoroughly rinsed 3 hours later by medical personnel. Petrolatum was applied topically and the wound bandaged. The patient continued the jungle race for the next 108 hours, during which the wound was hastily cleansed and rebandaged twice. The patient repeatedly swam in the Amazon Basin. At the race’s finish, the wound appeared purulent and was cleansed with tap water, soap, and a brush. Numerous attached ticks were removed from his skin.

The arm became tender, and the patient had bouts of intensified local pain. A possible, round entry wound, about 1 mm in diameter, was noted, at the bottom of which the patient observed motion. Five days after the accident, a physician explored the wound with scissors at a breakfast table. No larvae were found. Sugar was applied topically and cefalexine prescribed. Twenty hours later, the bouts of pain recurred with increasing frequency. The patient believed that something left in the wound “ate his flesh.” Nine days after the accident, at the Frankfurt Airport, Germany, the patient found a larva (Figure, part a) and an exit site wound (Figure, part b) under the bandage. The bouts of tenderness subsided. The patient’s wound was reexamined 1 day later and was found to be largely healed; the forming scar remained somewhat tender and itchy for 2 months. The maggot was sent to the Finnish Museum of Natural History, Helsinki, Finland, and identified as a third-stage larva of *Cochliomyia hominivorax* (Coquerel), the New World screwworm fly. In addition to the New World screwworm fly, an important Old World species, *Chrysoimya bezziana,* is also found in tropical Africa and Asia.

**Figure:**
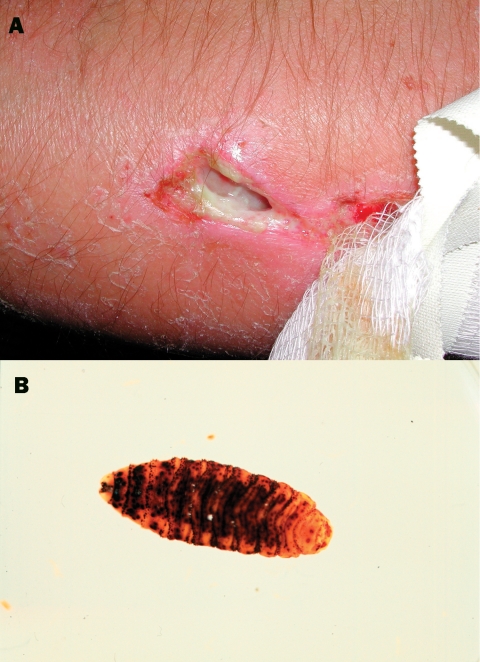
Exit site wound on patient’s arm (a) and a maggot, measuring 16 mm in length, from patient’s wound (b).

## Conclusions

Myiasis is the infestation of live humans and vertebrate animals by fly larvae. These feed on a host’s dead or living tissue and body fluids or on ingested food. In accidental or facultative wound myiasis, the larvae feed on decaying tissue and do not generally invade the surrounding healthy tissue (*1*). Sterile facultative *Lucilia* larvae have even been used for wound debridement as “maggot therapy.” Myiasis is often perceived as harmless if no secondary infections are contracted. However, the obligatory myiases caused by more invasive species, like screwworms, may be fatal (*2*). The screwworms are capable of penetrating through minor cracks in the skin, entering body openings, and migrating from wounds to living tissue. Most often this is initiated when the flies, attracted by the wounds, lay their eggs in necrotic, hemorrhagic, or pus-filled lesions. Infestation and penetration of the ocular, nasopharyngeal, paranasal, and auricular cavities are frequently described. (*3*). The female flies colonize the wounds with bacteria like *Providencia* spp., which in turn attract more flies by the distinct odor of the bacterial mass. Secondary bacterial infections are common. All conditions compromising the integrity of skin (like tick bites and skin diseases) predispose to the infestation (*4*). The eggs of *Cochliomyia hominivorax* are laid in batches. Hundreds of maggots of both sexes can be found in a wound. Screwworms can thus be introduced to new areas by just one index patient. Considerable risk exists of reintroducing *C. hominivorax* into areas from which it has been eradicated. Within 24 hours of hatching, the larvae begin feeding and cause extensive tissue destruction, pain, and even death. The larvae possess powerful oral hooks and can invade cartilage and bone (*5*). After feeding for 4 to 8 days, the larva leaves the wound to pupate in the soil (*6*,*7*). The pest is viable year round in areas with temperatures constantly >16°C.

*C. hominivorax* was first found to be infesting people in the penal colony of Devil’s Island in French Guiana in 1858. It was originally distributed from the southern United States to Argentina between the 35^o^ north latitude and the 35^o^ south latitude. Due to a massive sterile male fly release program**,** *C. hominivorax* was eliminated from North America. *C. hominivorax* is an important insect pest of livestock in the neotropical regions and has caused substantial losses to the livestock industries of the Americas (*7*). It is classified as a restricted animal pathogen in the United States. At present, populations of *C. hominivorax* are found in Central and South America and in certain Caribbean Islands (*4*,*6*,*7*). Human infestations remain an important health problem, primarily affecting the severely debilitated or persons with no access to healthcare. In the largest published study, the fatality rate was 2.8% (*7*). Isolated reports of infestations in the United States and Mexico are often traced to the importation of infested animals. Between 1969 and 1988, 39 human cases were reported (*7*). The potential for the introduction of *C. hominivorax* into new areas is ever present. In 1988, the screwworm appeared in Libya, most likely imported by infested livestock. It overwintered and became for the first time established outside the Western Hemisphere, where it infected 3,000 livestock and >200 persons. From Tripoli, it spread rapidly approximately 200 km and threatened to spread further to the savannas of sub-Saharan Africa. This possibility presented a potential threat of enormous proportions to the livestock, wildlife, and human populations of a large part of the Eastern Hemisphere. After a large-scale campaign, the screwworm was successfully eliminated from the area in 1991 (*7*,*8*).

In humans, the most common sites of *C. hominivorax* infestation are the nose, eyes, and skin. The manifestations depend on the anatomic region affected but are usually characterized by local pain, intense pruritus, cutaneous nodules, and larvae emerging from wounds or cavities (*7*,*9*). Massive infestations can result in the death of the host, usually attributed to “massive toxic shock” or to penetration of viscera or cavities, especially in the head and neck area (*6*,*8*). Infestation of paranasal sinuses often goes undetected for long periods. When infestation is suspected, a careful search for a larval infestation of eyes, nose, paranasal sinuses, or wounds should be performed, if necessary with the help of computed tomography or magnetic resonance imaging (*7*). Radiographic studies may only show edema. No effective antimicrobial therapy is available, although doramectin and ivermectin have been investigated for prophylactic use in cattle. Treatment involves removal of the larvae (reviewed in [*5*] and [*6*]). Irrigation with either chloroform or ether is advocated. Surgery is often required. For identification, the larvae should be hatched to adult flies, or first killed by immersion in nearly boiling water, then cooled and preserved in 80% ethanol. Secondary bacterial infections should be treated with local wound care and administration of antimicrobial agents.

In industrialized countries wound myiasis is a sign of neglected wound care, with mostly facultative myiases seen. The patients are often debilitated, of lower economic status, homeless, or substance abusers. The main focus is in treating secondary bacterial infections and in proper debridement. The risk of a traveler’s acquiring *C. hominivorax* is thought to be negligible (*3*). With the increasing popularity of adventure and wildlife travel, the risk may need to be reassessed. Travelers can contract aggressive obligatory myiases. This is the third report of *C. hominivorax* infestation in a tourist in 3 years (*4*,*9*). Also, a previous case was reported in a U.S. Army ranger who was wounded in action in Panama (*5*). In particular, medical personnel who treat patients who have participated in adventure sports events should recognize the intensity of the exposure to even the most exotic infectious diseases.
